# From Uganda to Baltimore to Alexandra Township: How far can Ainsworth’s theory stretch?

**DOI:** 10.4102/sajpsychiatry.v24i0.1137

**Published:** 2018-06-06

**Authors:** Nicola K. Dawson

**Affiliations:** 1 Department of Psychology, School of Human and Community Development, University of the Witwatersrand, South Africa

## Abstract

**Introduction:**

After extensive observation of mother-infant dyads in two diverse contexts, Ainsworth developed the construct of maternal sensitivity to explain the nature of mother-infant interactions that lead to infant attachment security. She believed this construct to be universally applicable. Since Ainsworth’s publications, her theory has been adapted and extended, particularly by theorists working in North American and Western European countries. These developments have been largely uninterrogated in relation to their universal cultural relevance, despite the fact that parenting practices differ greatly across cultural groups. Those who have begun to interrogate the cultural universality of current conceptualisation of maternal sensitivity highlight important areas of cultural disagreement.

**Method:**

This article provides a critical theoretical argument regarding the cultural universality of maternal sensitivity, extending comment to the cultural and contextual relevance of developments in its operationalisation.

**Results:**

Particular aspects of current theoretical and operational use of the construct of maternal sensitivity that are potentially culturally specific (as opposed to culturally universal) are noted, namely the inclusion of positive affect, the centrality of parent-infant play, verbal responsiveness, the inclusion of learning in parent-infant interactions and the shift towards a more proactive (rather than reactive) role for the parent in parent-infant interactions.

**Conclusion:**

This article suggests that the evolution of the concept of maternal sensitivity has failed to account for cultural differences.

## Introduction

Recent developments in the field of neuroscience have popularised attachment theory and clinicians across the globe are looking to attachment theory to help develop preventative mental health interventions and effective parenting strategies.^1,2,3,4,5^ Typically, such interventions attempt to promote positive child and societal outcomes through improving the quality of parent-infant attachment. Maternal sensitivity is a core construct in attachment theory that was developed to explain the parental qualities and behaviours that promote attachment security.^6^ Understandings of maternal sensitivity are, therefore, used to develop attachment-promoting interventions across the globe, and measures of maternal (or parental) sensitivity are extensively used to determine their success.^7,8,9,10,11^

While the global spread of theory and research is considered largely positive, failure to interrogate the applicability of imported knowledge can have negative repercussions. Most pertinently in the realm of attachment and early childhood development, the application of theory from ‘Western, educated, industrialised, rich, developed’ (WEIRD) contexts, without sufficient consideration of cultural and contextual difference, has resulted in an increasingly homogenous image of how children are or should be and what their childhood is or should be like.^13,14^ Pence and Nsamenang^13^ have labelled this the rise of the ‘global child’ and note the consequent negative implications for diversity and the potential for the pathologising of parenting customs in non-WEIRD contexts.

The Umdlezane Parent-Infant Programmes at the Ububele Educational and Psychotherapy Trust in Alexandra Township, Johannesburg, include a number of early interventions that aim to promote caregiver-infant attachment and healthy developmental trajectories, through increasing maternal sensitivity and supporting the parent-infant dyad. Interrogating the efficacy of these interventions is a central part of the programme’s work. During a study into the efficacy of the Ububele Home Visiting Project,^15^ the team undertook to code mother-infant interactions for maternal sensitivity using a Canadian-developed measure called the ‘Maternal Behavioural Q-Sort – mini’ (MBQS-mini).^16^ The training of the team in the measure and coding of the South African mother-infant interactions began to raise important questions about the use of imported measures in our local context.

The following excerpt is from the author’s coding notes, written while coding a video-recorded interaction between a 22-year-old South African mother, Thato,^1^ and her 5-month-old infant son, Tumi,^2^ for sensitivity using the MBQS-mini. The notes include clinical impressions post-coding:

After appearing initially unsure as to what is expected of her, mom has placed baby in front of her on the mat. Mom appears anxious, and is quiet and shy. Baby is vocalising. Mom watches, but does not respond physically or verbally. Mom notices the face on the top ring of the stacking toy. She picks it up and makes the stacking toy ‘walk’ over to baby. Baby takes stacking toy and mouths it. Mom prevents mouthing by removing it. Baby reaches for rattle and places it in his mouth. Mom prevents mouthing by removing rattle, but shakes it and gives it back. Baby drops rattle and it rolls out of baby’s reach. He signals that he wants it back. Mom picks up rattle and hands it back to baby, before looking anxiously back at the researcher behind the video camera. Baby reaches for ball, which is just out of reach. Mom notices this and taps ball closer to baby, who grasps it and bangs it on the floor with his left hand. Researcher hands book to mother. Mom turns pages for baby, allowing baby to touch and look. Still no verbalising from mother. Baby accidently knocks book closed. Baby takes closed book from mom and explores it … Baby is whining. After second whine mom puts baby on the breast. He drinks quietly.

Mom did not speak to baby once during this interaction, however she is responsive and baby appears regulated and content. Mom has lost points on MBQS-mini for failing to ‘facilitate learning’ in her interactions with the baby, for her lack of animation, her general lack of proactiveness and her use of objects and feeding to soothe. She has, however, allowed baby to explore the toys without intrusion and has let baby take the lead. She is certainly aware of her baby’s signals.

Mom discloses to the researcher at the end of the video that she felt shy during the video-recording. This ability to reflect on her own mental state in this anxiety-provoking situation is noteworthy, but I wonder about how scrutinised mom feels by the ‘white, professional researcher’. Regarding her use of the book and stacking toy, I wonder about mom’s level of literacy and her familiarity with such toys. Although mom scored in the moderate range, it doesn’t feel like the available items have captured her strengths.

Coding experiences like the one described above have been common amongst the coding team and have raised questions about the assumption of a ‘global mother’ alongside the ‘global child’, as represented in North American and Western European measures of sensitivity. Despite a great diversity in parenting practices across the globe,^12,17^ conceptualisations and measures of maternal sensitivity appear to provide a homogenised, WEIRD representation of parenting.

Interrogation into the cultural and contextual universality of the concept of maternal (or parental) sensitivity has begun. However, the literature is fairly disparate. This article attempts to bring together existing interrogations of the universal applicability of maternal sensitivity, while also expanding on critiques of current conceptualisations, contrasting them with anthropological literature. Beginning with an overview of Ainsworth’s seminal work on the concept of maternal sensitivity, the paper will go on to outline subsequent adaptations and revisions to the construct. This involves looking at both theoretical papers and papers on the operationalisation of the construct, with the aim of providing a clearer understanding of what we know about the universality of maternal sensitivity and what we have yet to understand.

## The development of the construct of maternal sensitivity

### Ainsworth’s construct of maternal sensitivity

The concept of maternal sensitivity emerged from the early work of American-born, Canadian-raised developmental psychologist Mary Ainsworth.^18^ Building on Bowlby’s^19^ attachment theory, Ainsworth et al.^6^ used the data from Ainsworth’s Ugandan and North American mother-infant observations to investigate how attachment patterns were formed and set out to describe and define the universal caregiving capacities and behaviours associated with attachment security, under the umbrella term ‘maternal sensitivity’.^6,18,20^

Ainsworth^6,18^ defined *maternal sensitivity* as the accurate interpretation and prompt and appropriate responsiveness to the full range (from overt to subtle) of an infant’s signals and communications. Maternal sensitivity was, and is, argued to be the main precursor to the development of attachment security.^6,19,21^ It is suggested that repeated sensitive interactions with a primary caregiver provide infants with an internal organising system that leads to the development of a secure base and, therefore, attachment security.^6,16^ Conversely, insensitive interactions were thought to lead to the development of insecure attachment.^6^

Ainsworth^6,18,22^ identified four core components necessary for the sensitive responsiveness to an infant’s signals: (1) awareness, (2) accurate interpretation, (3) appropriate responsiveness and (4) prompt responsiveness. At its core, maternal sensitivity is concerned with whether or not the mother supports or interferes with the activities that the infant initiates.^20,22^ It is not a predetermined list of sensitive parenting behaviours but rather a context-dependent, infant-dependent, sensitive response arising out of a psychological awareness of the infant.^22,23^ Ainsworth considered both her American and Ugandan observations in the development of her theory and therefore concluded that maternal sensitivity is a universal construct.^20^ She argued that, across all cultural variations, infants have a need for a trusted and sensitive attachment figure.^20^

### Theoretical adaptations of the construct

Since Ainsworth’s 1969 publication, the concept of maternal sensitivity has been significantly expanded upon and represented in a number of divergent ways in the literature.^23,24^ Maternal sensitivity is now used to describe a vast range of caregiving attributes and maternal behaviours.^24,25^ Shin et al.^24^ point out that maternal sensitivity is at times used interchangeably with the terms ‘parental sensitivity’, ‘maternal responsiveness’ and ‘maternal competency’. However, Shin et al.^24^ note that maternal responsiveness, defined as the ‘promptness or frequency of response to the infant’s signals’ (p. 309), is only one aspect of maternal sensitivity and lacks reference to the quality of the response in particular.^26^ They also make a distinction between maternal competence and maternal sensitivity, suggesting that maternal competence focuses more on skills and knowledge, rather than quality of response.^24^

Biringen^27^ has been a key proponent for the expansion of the concept of maternal sensitivity. In 2000, Biringen was a researcher at Colorado State University in the United States. While developing a scale of emotional availability, Biringen^27^ proposed that conflict resolution and emotional tone should be considered central aspects of (maternal) sensitivity. Biringen^27^ argued that a sensitive mother displays a positive affect and is warm, flexible and able to soothe her distressed infant. While Biringen^27^ provides evidence from her own research sample in support of her argument, the universal validity of these extensions are uncertain. The view that warmth and positive affect are central components of maternal sensitivity contrasts with Ainsworth’s stance, that positive affect or ‘maternal warmth’ did not appear to be universally expressed and seemed to have minimal impact on attachment.^20^

Meins et al.^23^ also advocated for the rethinking of maternal sensitivity, suggesting that Ainsworth’s original definition lacked clarity. At the time of publication, the authors were working at Staffordshire University in the United Kingdom. The group suggested that the concept of mind-mindedness – the mother’s ability to read her infant’s mental states – provided a clearer focus than Ainsworth’s conceptualisation of maternal sensitivity. Mind-mindedness is considered to be an extension of Ainsworth’s understanding that the sensitive mother is able to read her infant’s signals accurately and respond appropriately. The group argued that while Ainsworth’s description captures a mother’s general sensitivity to her infant’s physical and emotional needs, the construct of mind-mindedness looks specifically for sensitivity to the infant’s mental states and ongoing activity, even when the infant’s emotional and physical needs are satisfied. Here the group advocates for the need for sensitivity even in the absence of signals or needs, in contrast to Ainsworth’s understanding. Meins et al.’s^23^ argument centred around a study of 71 low- to middle-class mother-infant dyads, who all appear to be from the United Kingdom.

### Adaptations related to operationalisation

Ainsworth^18^ published her construct of maternal sensitivity alongside a tool for its measurement. The Ainsworth tool^18^ is comprised of a global nine-point scale, with descriptors ranging from highly sensitive (score of 9) to highly insensitive (score of 1). In developing this tool, Ainsworth noted that she took into account her observations in both Baltimore and Uganda, highlighting that while there was great similarity in parenting practices across the two contexts, there were also important differences, such as the difference in displays of warmth and positive affect noted above.^20^

Since the development of Ainsworth’s^18^ scale, a number of additional related measures have been developed, the most prominent of which were identified and reviewed by Mesman and Emmen.^22^ Of those identified, five were developed in the United States: the CARE-Index,^28^ the Emotional Availability Scales,^29,30^ the Erickson scales,^31^ the NICHD-SECCYD sensitivity scales^32^ and the Parent-Child Early, Relational Assessment.^33^ An additional measure, the MBQS, was developed in Canada.^16^ The supporting data for these measures appears to have been predominantly collected in North America, with experts from the same region contributing to the development of the measures.^22,31,34,35^ A sixth measure, called the ‘Global Ratings of Mother-Infant Interaction’, was developed in the UK, drawing on data from a sample of mothers from Cambridge.^36^ Only one scale was developed outside of Northern America or Western Europe, the measure entitled ‘Coding Interactive Behavior’ (CIB), which was developed in Israel.^37^

There has been great variation, divergence and deviation from Ainsworth’s understanding of maternal sensitivity in the development of these alternative measures.^22^ In these scales, sensitivity scores incorporate dimensions of parenting not originally explicitly understood to form part of maternal sensitivity, including the above-mentioned ‘conflict resolution’ and ‘positive affect’ (see Emotional Availability Scales), as well as ‘clarity of instructions’ and ‘respect for autonomy’ (see Erickson scales) and ‘facilitation of exploration and learning’ (see MBQS).^22^ In their review, Mesman and Emmen^22^ highlighted three core deviations from Ainsworth’s original construct across a number of the measures listed above, including (1) observation of interactions in a structured play setting rather than in naturalistic settings, (2) the inclusion of positive affect and (3) the use of composite scales or domains of sensitivity, rather than a single global scale.

The process of operationalisation has also often resulted in the explicit description of predefined ‘sensitive’ or ‘insensitive’ behaviours.^38^ For example, behaviours stated as indicative of sensitivity on the MBQS include ‘notices when baby smiles and vocalises’ as well as ‘praises baby’.^39^ In the Emotional Availability Scale, discrete behaviours are also looked for, including the mother’s ability to find interesting, stimulating and creative ways to play with her infant.^27^ This greater specificity as to the type of infant signals a mother should notice and how she should respond diverges from Ainsworth’s more broad and abstract definition and scale descriptors. However, it has been argued that Ainsworth’s more abstract descriptions provided little clarity,^23^ leaving the complex task of analysing human social interaction too open to subjectivity. Therefore, attempts to operationalise the construct by linking it to predefined behaviours is understandable and probably motivated, in part, by attempts to prevent subjectivity and bias in observers or coders. However, the opposite may actually have occurred, by introducing cultural bias in the behaviours that are specified. It is the position of this article that broader descriptions may allow for (and in Ainsworth’s case, may intentionally have served to allow for) contextual and cultural variance in expressions of maternal sensitivity.

## Ethical Consideration

Permission was obtained from both the mother mentioned in the paper and the Ububele Educational and Psychotherapy Trust, to make use of data obtained as part of a randomised control trial in this publication. The name of the mother and infant mentioned in this paper has been changed to protect their identity.

## Thinking critically about ‘maternal sensitivity’

### An emerging debate

While the concept and operationalisation of maternal sensitivity has been revisited extensively over the last 50 years, the developments have occurred almost exclusively in WEIRD countries in Northern America and Western Europe.^40^ Furthermore, very few authors writing on the construct have addressed the issue of cultural and contextual applicability or variation. However, on review of the literature, evidence of thinking about contextual and cultural relevance can be found, starting covertly at first and moving towards a current emerging debate.

As far back as 1981, references to ‘parental sensitivity’ (as opposed to maternal sensitivity) are observed.^41^ And the use of this more gender-inclusive term has become more commonplace over time.^27,40,42^ Although never overtly stated, the shift from the use of the term ‘maternal sensitivity’ to ‘parental sensitivity’ challenged a cultural and contextual bias inherent in the initial construct – that the mother is the primary caregiver and that her interactions with the child exclusively determine the child’s attachment style. Subsequent studies have demonstrated the importance of sensitive father-child interactions in child development.^43,44,45^ This is the first evidence of a cultural challenge to the construct.

Far more recently, an enquiry into the cultural universality of the construct of maternal sensitivity and its measurement has been emerging, predominantly from South Africa and the Netherlands.^46,47,48,49,50,51^ While some support is being found for the qualities of a ‘global mother’, there may be cultural variation in the activities undertaken by mothers in order to achieve the same aims. Mesman and Cisse^47^ investigated whether or not the sensitive responsiveness of caregivers, as measured by the Ainsworth scale, was compatible with local caregiving standards in Mali and concluded that Ainsworth’s concept of sensitivity and parenting norms in Mali may have some common ground. Mesman et al.^49^ also investigated whether maternal beliefs about the ideal mother converged with the ideal mother depicted in the MBQS across varied cultural contexts in 26 countries. The study found strong convergence between maternal beliefs and the MBQS’s ideal mother, suggesting that the basic tenets of the concept of maternal sensitivity have universal relevance. However, the use of the MBQS-mini in a South African study^15^ raised questions amongst the South African coding team, who found that South African mothers tended to score poorly on the MBQS-mini, primarily because of low scores on items noting warm positive affect, facilitation of learning, praise and verbal responsiveness.^46,51^ This suggests that while mothers may agree on the description of a sensitive mother cross-culturally, how this sensitivity manifests in the behaviours they exhibit may vary. The latest literature suggests that recent conceptualisations of maternal sensitivity have left little room for cultural variation.^48^ In particular, this article suggests that verbal responsiveness and positive affect are interactions more specific to Western contexts, while more subtle forms of responsiveness (including physical facilitation) are more common modalities for sensitive responsiveness in many non-Western contexts.

Lastly, the relevance of the construct of maternal (and even parental) sensitivity for contexts where a network of multiple caregivers (alloparenting) is the norm (such as the northern Philippines) was also raised in 2016.^48^ Mesman et al.^50^ proposed that the concept of ‘received sensitivity’ served as a more universally appropriate conceptualisation of maternal sensitivity and sensitive responsiveness.

### Areas of possible cultural bias

An interrogation of the psychological literature, when compared with anthropological literature on parenting, reveals a number of areas of possible cultural bias. This critical analysis of the literature revealed potential shifts away from cultural universality in five key areas.

#### The inclusion of positive affect, warmth and affection

As noted previously, various theoretical arguments as well as measures of maternal sensitivity have included expression of genuine positive affect, warmth and affection as a central factor in sensitive responsiveness.^21,27,36,37,52^ This is the first area of adaptation where cultural specificity is arguably at play. In the coding excerpt given above, Thato showed no expressions of positive affect, warmth or affection towards Tumi, yet she consistently, promptly and contingently met each of Tumi’s signals.

Arguments questioning the universal cultural applicability of positive affect, warmth and affection as a central component of maternal sensitivity already exist in the literature. As noted above, Mesman and Emmen^22^ identified the inclusion of positive affect as a key divergence from Ainsworth’s original construct. In noting this, they proposed that positive affect may be a culturally specific adaptation to the construct and argued that, in fact, high levels of positive affect are often accompanied by high levels of intrusiveness and lack of signal perception. They suggested that positive affect may, therefore, actually be at odds with central components of maternal sensitivity.

Anthropological literature supports the idea that the importance of expressions of warmth and positive affect is culturally specific. The literature shows that there is great variation in the intensity with which emotions are expressed across cultural groups.^53,54^ In addition, Ainsworth^20,55^ herself ruled out warmth and affection as a central component of maternal sensitivity. Ainsworth felt that warmth and positive affect were important in her Baltimore study but did not find the same in all of the Ugandan mothers. It should, however, be noted that in a study by Mesman et al.^49^ there were high levels of agreement amongst 26 Western and non-Western cultural groups that positive affect and warmth are attributes of an ideal mother. Similarly, there was agreement that the inverse (negative or flat affect) was one of the least ideal caregiving attributes. While this may simply reflect that expressions of positive affect are universally viewed as more favourable than expressions of negative affect, perhaps the required frequency of these affective displays differs. While the absence of positive affect may be significant in many of the North American contexts where this feature was added, there is insufficient evidence to suggest that it is appropriate to all contexts or, more importantly, a universally necessary component of maternal sensitivity.

#### The assumption of caregiver-infant play as a naturalistic interaction

The second area where the development of the construct of maternal sensitivity may have moved us away from cultural universality is the area of play. Play has become central to much of the modern use of the construct of maternal sensitivity in a number of ways. Firstly, play-based interactions have become synonymous with measures of maternal sensitivity and sensitive responsiveness. In Mesman and Emmen’s^22^ systematic review of measures of maternal sensitivity, ‘free play’ was identified as the most commonly used observational setting, with a high presence of more structured play settings as well. This diverges from Ainsworth’s call to focus on naturalistic observation when measuring maternal sensitivity.^20^ While mother-baby play interactions appear to be very common in North America and many European settings, anthropological studies show that this is not true of all contexts. Lancy^56^ argues that there has been an inaccurate assumption by psychologists that mother-infant play is a familiar and natural occurrence for mother-infant dyads the world over. Rather, Lancy^56^ argues that mother-child play is a culturally specific phenomenon. This is supported by other researchers. Levene et al.^57^ argue that, in Africa, mothers are not considered appropriate playmates. Rather, play with the infant is the task of siblings and other children. A lack of familiarity with mother-infant play interactions may explain some of Thato’s hesitancy and awkwardness in the excerpt described above.

In addition to the use of play as an assumed naturalistic interaction, the ability to play with an infant and the skill of the adult as playmate has been incorporated into some definitions and measurements of maternal sensitivity, as noted above. Various measures of maternal sensitivity have added play as a key criterion for a high maternal sensitivity score.^27,58,59^ More specifically, the measure of Emotional Availability scores the mother with regard to the amount of play in which she engages, the type of play she initiates and the extent to which the play appears to be fun. The universal cultural relevance of these skills with regard to the understanding of a mother’s capacity for sensitive responsiveness must be questioned, given that mother-infant play is not universally practised. Further, there is no evidence to suggest that, universally, the parent’s skill as a playmate has consequences for the attachment system.^17,60^ Rather, it is the mother’s responsiveness to the infant’s bids for interactional activity that seem relevant. Thato would score poorly as a playmate for Tumi, failing to get involved in or initiating mutual play. Yet her inability to play seems to have no bearing on her sensitive responsiveness shown throughout the excerpt.

Lastly, toys such as books and puzzles are often introduced as part of the standardised setting for observing interactions in the majority of measures of maternal sensitivity. Some test items also show interest in the mother’s use of toys. The 90-item MBQS has as one of its items ‘provides age appropriate toys’.^21^ In many contexts, children do not have access to toys but rather create their own objects for play. Items such as the one on the MBQS display various context-specific ideas regarding infant play, such as that play involves the parent, that play involves objects and that objects for play are provided to the infant by adults rather than discovered by the infants themselves. These assumptions are made from contexts where play has been commercialised through the mass production of toys. Further, it can also be argued that no toys are universally available and that therefore they should not form part of universally relevant measures of maternal sensitivity. Thato is clearly unfamiliar with the stack toy and doesn’t realise its function. She also largely leaves Tumi to discover and engage with the toys himself. Again, this seems to have no bearing on her sensitive responsiveness.

The reliance on play and toys, as described above, has clear implications for the universal applicability of measures of maternal sensitivity. The use of standardised toys or play-based situations across all cultural contexts is problematic. The majority of mothers from cultures where play between adults and children is not common, when asked to engage in play with their infant in order to measure their sensitivity, are disadvantaged, as they find themselves engaging in an unfamiliar activity, while under researcher scrutiny. Maternal sensitivity scores will be differently impacted across contexts by the mother’s familiarity with the toy and with play, calling the universality of such measures and understandings into question.

#### The central role of language, verbalisations and verbal responses

It is noted above that more recent measures of maternal sensitivity have moved away from abstract notions of responsiveness towards descriptions of specific behaviours that are considered sensitive.^38^ In particular, language, verbalisation and verbal responsiveness have been introduced as significant components of maternal sensitivity, as preferential over physical forms of response. For example, mothers lose points on the MBQS-mini if their interactions are ‘physical rather than verbal’ or ‘object oriented’. The CARE-Index scores parents according to their use of vocal expressions.^28^ The CIB also looks at the quality of vocal interactions.^37^

This preference for verbal responsiveness has been identified as a third area of cultural-specific adaptations in the literature, with clear implications for silent yet responsive mothers like Thato. Although Thato does not speak to Tumi once throughout her interaction with him, and often responds to his signals physically or with objects (such as toys or the breast), these responses always seem to be not only sufficient but appropriate and contingent.

Linguistic anthropology has long concluded that some cultural groups are more verbally communicative than others.^61,62,63^ In particular reference to parent-infant interactions, anthropology has demonstrated that talking to preverbal infants is not a universal phenomenon.^62^ There is also extensive evidence that suggests that different cultural groups make use of different modalities to respond to infants’ signals. ^22,38,59,64,65,66,67,68,69,70^ While Western parents tend towards more overt, verbal forms of interaction, non-Western parents’ interactions appear to be more subtle and non-verbal.^38^ Active social and extroverted behaviours such as face-to-face positioning, eye contact and vocalisation are also more commonly found in North American and Western European cultures. In contrast, parents in African, Asian or South American cultures are commonly found in continuous close physical proximity to their infant and appear to primarily make use of physical facilitation, focus-following, tempo adjustment, movement and positioning in response to infant signals.^48,49^ Such parents are unlikely to engage in normal speech or motherese (baby talk), eye contact or face-to-face interaction. Given these cultural variations, many groups are likely to be misrepresented by current conceptualisations or measures of maternal sensitivity, which consider vocal interactions to be central. This evolution towards an emphasis on verbalisation is interesting given Stern’s ^71^ assertion that response to infant cues may happen in many modalities and that cross-modal responses are actually thought to have developmental benefit.

#### The inclusion of learning in parent-infant interactions

The parent’s role in the facilitation of the infant’s learning has also become intimately linked with theoretical arguments about and measures of parental sensitivity and sensitive responsiveness. The parent’s ability to facilitate learning has come to be considered as a domain of sensitivity, either in a separate composite scale or as part of a global sensitivity scale. The MBQS-mini, for example, notes that the ‘facilitation of learning’ is a core domain of the measure and describes the sensitive mother as one who ‘creates and encourages an environment conducive to learning and exploration’ and ‘structures the environment and interactions to promote learning’.^72^ Biringen’s^29^ measure of emotional availability is also interested in the mother’s ability to structure activities and create boundaries. Erickson et al.’s^31^ measure focuses specifically on the learning setting and considers adults’ provision of clear instructions, their clarity of instructions and their respect for the child’s autonomy as central components of a high score. Further, the NICHD-SECCYD sensitivity scales have a special focus on sensitivity during teaching tasks.^31^

A focus on learning during parent-child interactions clearly steps away from Ainsworth’s core understanding of maternal sensitivity – the prompt and appropriate response to an infant’s signals. A focus on learning is, at best, a separate issue, still relevant to child development. However, its universal relevance is highly questionable. Anthropological research demonstrates that across the majority of cultural groups parents have little active involvement in their child’s learning.^73,74,75^ Lancy and Grove^74^ note that even in the West, where parenting is largely synonymous with teaching (in contrast to the rest of the world), this is a recent development. Infant learning is more widely understood to be dependent on ‘their natural curiosity and motivation to emulate’ experts, as well as their interactions with other children.^73,75^

Such anthropological literature supports the argument that, through the increased inclusion of ‘facilitation of learning’ as a key component of parental sensitivity and sensitive responsiveness, understanding and measurement of maternal sensitivity has become more and more culturally specific. Applying this understanding and measurement in contextual settings where a focus on learning through parent-infant interaction is not the norm could strongly disadvantage parents like Thato, who makes no attempt to teach or facilitate learning. Although attachment security is known to have implications for learning,^76^ there is no clear evidence to suggest that facilitation of learning in parent-child interactions is a universally important component of maternal sensitivity or sensitive parent-infant interactions more generally, and thus it should not be put forward as universally applicable.

#### Proactive rather than reactive adult involvement in interactions

Ainsworth’s original conceptualisation of maternal sensitivity is a reactive one. Sensitivity is conceptualised as a response to a signal that originates with the infant. In the absence of a signal from the infant, no action is necessary from the mother/parent. For Ainsworth, maternal sensitivity is concerned with whether the mother supports or interferes with the things that the infant initiates.^20,22^ If the infant does not signal, nothing is required of the mother, other than to refrain from intruding or interfering.

A close reading of the subsequent literature on maternal sensitivity reveals a shift in this position. Later conceptualisations and measures increasingly require the mother to be proactive and to act even in the absence of a signal. For example, the MBQS-mini describes how a mother must proactively ‘encourage … exploration’, ‘create … an environment’, ‘structure an environment’ and ‘promote and initiate interactions …’.^72^ As one of their core sensitive items, the measure is also interested in whether the mother ‘builds on the focus of baby’s attention’. As part of the Emotional Availability Scales, a mother needs to find interesting, stimulating and creative ways to play with her infant to score highly.^27^ In addition, the concept of mind-mindedness purports that sensitivity to the infant’s mental states and ongoing activity is necessary even when the infant’s emotional and physical needs are satisfied and the infant does not signal. Such descriptors require a far more active position from the mother, even in the absence of signals (distress and non-distress). From such a position, a more watchful, less actively involved (but still sensitively responsive) mother, such as Thato, is considered less sensitive and viewed more negatively. Yet it is clear that, from her passive, watchful position, she responded to Tumi promptly and sensitively. Thato’s failure to be proactive for most of her videoed interaction with Tumi yet again seems to have no bearing on her sensitive responsiveness to him.

## Conclusion

Maternal sensitivity appears to be a crucial concept for understanding attachment and promoting infant mental health and attachment security. However, this article has tracked how theoretical advancement and operationalisation of the construct of maternal sensitivity has taken place predominantly in developed countries, potentially opening the doors for cultural bias. This article suggests that the evolution of the concept of maternal sensitivity has failed to account for cultural differences, where parenting beliefs, social goals, parenting strategies, caregiving behaviours, intensity of emotional expression and relationships differ.^17,38,55,65,73,77^ Consequently, an image of the ‘global mother’, which fails to allow for benign difference and diversity and potentially pathologises other parenting practices, dominates. This has occurred, in particular, through the elevation of interactions that include positive affect, adult-infant play, verbal responses, opportunities for learning and proactive adult involvement.

Whether Ainsworth’s original conceptualisation and model is more culturally universal remains debateable. Its broad and abstract nature does leave room for subjectivity. However, attempts to remedy this through the inclusion of more specific descriptions of ‘sensitive behaviour’ appear to have made the construct even more vulnerable to cultural and contextual bias, values and assumptions. Caution must be exercised in the development of sensitivity-promoting interventions, and the results generated by more specific measures of maternal sensitivity should not be assumed to be generalisable or universally relevant. With cultural and contextual variation in parenting practices, goals and modalities, as well as variation in ideas of optimal child development, the understanding and measurement of maternal sensitivity across diverse populations is a difficult and ethically challenging endeavour that requires contextual consideration.

## Footnotes

1 Name has been changed.

2 Name has been changed.
